# Dietary Tomato Pectin Attenuates Hepatic Insulin Resistance and Inflammation in High-Fat-Diet Mice by Regulating the PI3K/AKT Pathway

**DOI:** 10.3390/foods13030444

**Published:** 2024-01-30

**Authors:** Jing Sun, Kongyan Wu, Pan Wang, Yubin Wang, Dan Wang, Wenting Zhao, Yuanyuan Zhao, Chunhong Zhang, Xiaoyan Zhao

**Affiliations:** 1 College of Food Science, Shenyang Agricultural University, Shenyang 110866, China; sunjing7353@163.com (J.S.); 17737816262@163.com (K.W.); 2 Beijing Key Laboratory of Agricultural Products of Fruits and Vegetables Preservation and Processing, Key Laboratory of Vegetable Postharvest Processing, Ministry of Agriculture and Rural Affairs, Institute of Agri-Food Processing and Nutrition, Beijing Academy of Agriculture and Forestry Sciences, No. 50 Zhanghua Street, Haidian District, Beijing 100097, China; wangpan@iapn.org.cn (P.W.);

**Keywords:** tomato pectin, insulin resistance, PI3K/AKT pathway

## Abstract

Chronic metabolic disease is a serious global health issue, which is accompanied by impaired insulin resistance. Tomato pectin (TP) is a naturally soluble complex hetero-polysaccharide with various biological functions. However, the impact of TP on hepatic insulin resistance in a high-fat diet (HFD) and its potential mechanism remains largely unknown. The results revealed that TP treatment significantly decreased the liver weight, hepatic fat accumulation and hepatic injury in HFD-fed mice. TP also improved fasting blood glucose levels and glucose tolerance in HFD-fed mice. The underlying mechanisms involved in the inflammation, oxidative stress and insulin signaling in the liver were also investigated by RT-qPCR and western blot, which indicated that TP ameliorated hepatic insulin resistance by regulating the PI3K/AKT/GSK-3β pathway, increasing the expression of GLUT4, decreasing the expression of PECK and G6P as well as restoring antioxidant activities and suppressing the inflammation statues in HFD-fed mice. Our data showed that dietary TP has profound effects on hepatic insulin resistance, inflammation and oxidative stress, demonstrating that TP might be a promising therapeutic agent against insulin resistance and related chronic metabolic disease.

## 1. Introduction

Chronic metabolic disease has become a serious health problem, representing one of the key causes of morbidity and mortality worldwide [1]. Obesity, non-alcoholic fatty liver disease and type Ⅱ diabetes (T2DM) are common chronic metabolic diseases, characterized by the dysregulation of glucose and lipid metabolism. The liver tissue is responsible for regulating systemic glucolipid metabolism homeostasis. Once the balance is broken, it could bring out inflammation and oxidative stress, followed by systemic insulin resistance [2]. Insulin resistance, metabolism and inflammation are strongly interconnected, which has been proven in human samples and in animal models. The dysregulation of hepatic glucose metabolism in T2DM patients begins with lipid-induced hepatic insulin resistance [3]. Insulin resistance-induced increase in hepatic lipogenesis further causes liver steatosis in obese subjects. Most treatment for chronic metabolic disease is aimed at using insulin sensitizers to protect hepatocytes from further damage [4]. Hence, controlling liver insulin sensitivity offers a viable strategy for counteracting chronic metabolic disease.

The key aspects in the development of chronic metabolic disease are the presence of tissue inflammation, enhanced oxidative stress and increased fat accumulation; these are linked through insulin resistance. The association between inflammation and insulin resistance is well known, but the mechanism of this association is not completely understood. The phosphoinositide 3-kinase/protein kinase B (PI3K/AKT) signaling was known as a major pathway of insulin resistance. In the liver, insulin binds to its receptor, promoting insulin receptor substrates insulin receptor substrate-1 (IRS-1) and insulin receptor substrate-2 (IRS-2) tyrosine phosphorylation, activating PI3K and Akt, which regulate glucose transport and metabolism [5]. Notably, inflammation and oxidative stress contribute to the progress of metabolic-related insulin resistance. Proinflammatory cytokines secreted from adipocytes not only promote insulin resistance of adipose tissue but also affect other tissues through the circulation transportation, such as liver. Hepatic insulin resistance was observed with the elevated level of tumor necrosis factor-α (TNFα) via decreasing the insulin signaling and glucose transporter type 4 (GLUT4) expression [6,7]. Moreover, disruption of hepatic insulin signaling by Interleukin- 6 (IL6) has also been found in vitro cell lines of liver. The knockout of monocyte chemotactic protein-1 (MCP1) improved insulin resistance in HFD-fed mice [6]. Previous studies also showed the change in oxidative stress state was related to enhanced inflammation and played a considerable role in the process of insulin resistance development [8]. High levels of reaction oxygen species (ROS) are formed in mitochondria to accelerate DNA damage and consequently, to increase insulin resistance. Compared to the control group, the insulin resistant mice have a higher malondialdehyde (MDA) and a lower catalase (CAT) and superoxide dismutase (SOD) activity [9,10]. Considering that, attenuated inflammation and oxidative stress might be a potent preventative strategy to alleviate insulin resistance and related metabolic disease [11]. 

Natural products could be considered as a supportive tool to keep patients with chronic metabolic disease healthier, including decreased proinflammatory cytokine expression, reduced insulin sensitivity, regulation of lipid metabolism and decreased body weight. Pectin, a naturally soluble complex hetero-polysaccharide, exhibits various biological functions, such as anti-obesity, anti-nonalcoholic fatty liver disease (NAFLD), anti-lipidemia, anti-inflammation and antioxidative. Pectin consumption of 3.3%, 6.7%, and 10% were negatively related to food intake and body weight gain in HFD-fed rat models. Among the various types of pectin, the function of apple pectin and citrus pectin has been explored extensively. Apple pectin has been reported to alleviate HFD-induced obesity, attenuate liver steatosis and decrease the level of TNFα and IL6 [12,13]. Citrus pectin administration in HFD-fed mice exhibited lower body weight and liver weight, and improved lipid and glucose metabolism. Other types of pectin have also been investigated in recent years. Bagabaldo et al. found that banana pectin ameliorated HFD-induced obesity by cholesterol and bile acid binding pathways [14]. Jiao et al. reported that ginseng berry pectin improved lipid disorder in obese rats through regulating gut microbiota and activating the AMP pathway [15]. Interestingly, tomato pectin (TP) is a potential active ingredient in tomato processing residue. Nevertheless, the effect of TP on chronic metabolic disease, especially on insulin resistance, inflammation and oxidative stress in HFD-fed mice remains unclear.

In this study, we utilized an HFD-induced chronic metabolic disease model to explore a possible connection between the TP treatment and HFD-associated derangements. We found that the effect of TP administration on hepatic insulin response inflammation and oxidative stress, thus contributing to improving the glucose metabolism in HFD-fed mice.

## 2. Materials and Methods

### 2.1. Preparation and Extraction of TP

The tomato pomace was obtained from Tunhe Tomato Co., Ltd., Xinjiang, China. TP was prepared by acid extraction according to the method described by Yang et al. with slight modification [16]. The tomato pomace was mixed with hydrochloric acid (HCl) extraction solvent (85 °C, pH 1.4) in a solid-to-liquid ratio of 1:50, and then extracted for 90 min at 85 °C using a reciprocating shaker bath (200 rpm). The hot mixture was centrifuged at 10,000 rpm for 20 min at 18 °C. The supernatant was collected and precipitated using two volumes of anhydrous ethanol and kept overnight without stirring.

### 2.2. Animals and Treatment

The animal study was approved by the Biomedical Ethical Committees of Peking University (Beijing, China) according to the guidelines, with the approval number LA2021305. A total of 24 six-week-old C57BL/6J male mice (Beijing Vital River Laboratory Animal Technology Co., Ltd., Beijing, China) were housed (four mice per cage) in a standard specific pathogen-free (SPF) facility with light/dark cycles of 12 h/12 h at 24 ± 2 °C, and 45 ± 5% humidity. 

**Figure 1:**
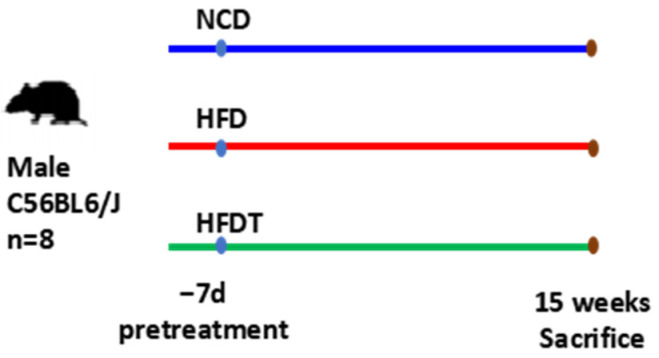
Experiment design. (Abbreviated: NCD: Normal control diet; HFD: High-fat diet; HFDT: High-fat diet administered with tomato pectin).

The experiment design is shown in Figure 1. After 1 week of adaptive feeding, mice were randomly subdivided into 3 groups (8 per group) that were treated for 15 weeks: normal control diet (NCD, 10% dietary fat) group; high-fat diet (HFD, 60% dietary fat); high-fat diet administered with TP (HFDT, 400 mg/kg/day tomato pectin by gavage, tomato pectin dry powder dissolved in saline (equivalent to 44 mg/kg/d for human). The NCD and HFD groups were given an equal volume of normal saline as controls. The diet ingredients and energy densities used in our study are shown in Supplementary Table S1 (Research Diets, Inc., New Brunswick, NJ, USA). From the beginning of the experiment, food intake and body weight (BW) were weighed twice per week. At the end of the experiment, the animals were euthanized after anesthetizing with ether by cervical dislocation. Serum and liver tissues were harvested for further analysis.

### 2.3. Biochemical Analysis

Serum total cholesterol (TC), triacylglycerol (TG) and glucose were tested using a blood biochemistry analyzer. Serum insulin was measured using the mouse insulin ELISA kit (ALPCO, Salem, NH, USA). Serum adiponectin (AdipoQ), leptin and free fatty acids (FFAs) and oxidative stress parameters in hepatic like total antioxidant capacity (T-AOC), glutathione (GSH), SOD, MDA and robot operating system (ROS) were tested using the related kits (Nanjing Jiancheng Bioengineering Institute Co., Ltd., Nanjing, China). All kits are used strictly according to the manufacturer’s instructions. Homeostasis model assessment-estimate insulin resistance (HOMA-IR) was calculated according to the following formula:

HOMA-IR = serum glucose (mmol/L) × serum insulin (mU/L)/22.5

### 2.4. Glucose (GTT) and Insulin Tolerance (ITT) Tests

The setup of this study closely followed our previously described method [17]. 

### 2.5. Quantitative Real-Time PCR (qRT-PCR) Analysis

Total RNA of the liver and ileum was isolated using Trizol (Thermo Fisher Scientific, Haverhill, MA, USA) and purified using an RNeasy Mini Kit (Qiagen, Frederick, MD, USA). RT-PCR was performed using an SYBR Green and the LightCycler 480 Real-Time PCR system (Roche Diagnostics, Shanghai, China). The primers sequences were as the following: IL-1β (IL-1β-F: 5′-GCAACTGTTCCTGAACTCAACT-3′; IL-1β-R: 5′-ATCTTTTGGGGTCCGTCAACT-3′), MCP1 (MCP1-F: 5′-TTAAAAACCTGGATCGGAACCAA-3′; MCP1-R: 5′-GCATTAGCTTCAGATTTACGGGT-3′), TNF-α (TNF-α-F: 5′-GACGTGGAACTGGCAGAAGAG-3′; TNF-α-R: 5′-TTGGTGGTTTGTGAGTGTGAG-3′), IL6 (IL6-F: 5′-TAGTCCTTCCTACCCCAATTTCC-3′; IL6-R: 5′-TTGGTCCTTAGCCACTCCTTC-3′); IL10 (IL10-F: 5′-GCTCTTACTGACTGGCATGAG-3′; IL10-R: 5′-CGCAGCTCTAGGAGCATGTG-3′), G6P (G6P-F: 5′-TCTGTCCCGGATCTACCTTG-3′; G6P-R: 5′-GAAAGTTTCAGCCACAGCAA-3′); GLUT4 (GLUT4-F: 5′-GGCTTTGTGGCCTTCTTTGAG-3′; GLUT4-R: 5′-GACCCATAGCATCCGCAACAT-3′), GS (GS-F: 5′-GGATGAATTCGACCCCGAGA-3′; GS-R: 5′-GTCACCTTCGCCTTCGTCTG-3′), GSK-3β (GSK-3β-F: 5′-GTAGCCCAGGGAGGTCACTA-3′; GSK-3β-R: 5′-ACCATCTCCAGCCTCATCCT-3′), INSR (INSR-F: 5′-ATGGGCTTCGGGAGAGGAT-3′; INSR-R: 5′-GGATGTCCATACCAGGGCAC-3′), IRS1 (IRS1-F: 5′-CGATGGCTTCTCAGACGTG-3′; IRS1-R: 5′-CAGCCCGCTTGTTGATGTTG-3′), PEPCK1 (PEPCK1-F: 5′-ATCATCTTTGGTGGCCGTAG-3′; PEPCK1-R: 5′-CATGGCTGCTCCTACAAACA-3′). The expression of each gene was normalized to glyceraldehyde-3-phosphate dehydrogenase (GAPDH). Relative quantification was performed according to the 2^−ΔΔCt^ method.

### 2.6. Western Blot Analysis

Frozen mouse liver tissues were homogenized in radioimmunoassay (RIPA) buffer (Beyotime, Shanghai, China), consisting of 30 mM Tris (pH 7.5), 150 mM Sodium chloride (NaCl), 1% Nonidet P-40 (NP-40), 0.5% sodium deoxycholate, 0.1% Sodium dodecyl sulfate (SDS), 10% glycerol and 2 mM Ethylene Diamine Tetraacetic Acid (EDTA), and containing the Complete Protease Inhibitor Cocktail (ThermoFisher Scientific, Waltham, MA, USA). The concentrations of protein were measured using the bicinchoninic acid assay (BCA) Protein Assay Kit (Solarbio, Beijing, China). Then, the proteins were separated by SDS-PAGE, and transferred onto nitrocellulose membranes (Bio-Rad, Hercules, USA) by electroblotting, blocked with 5% skimmed milk for 1 h and incubated with specific antibodies listed in Supplementary Table S2. 

### 2.7. Statistical Analyses

All data are reflected as mean ± SEM. GraphPad Prism (GraphPad Software 5.0) was used for graphing and statistical analysis. Statistical significance was analyzed by unpaired Student’s tests or one-way ANOVA followed by Tukey’s post hoc test for two or multiple comparisons and statistically significance was accepted at *p* < 0.05. was considered statistically significant. 

## 3. Results

### 3.1. Effect of TP on Body Weight and Liver Index in HFD-Fed Mice

**Figure 2:**
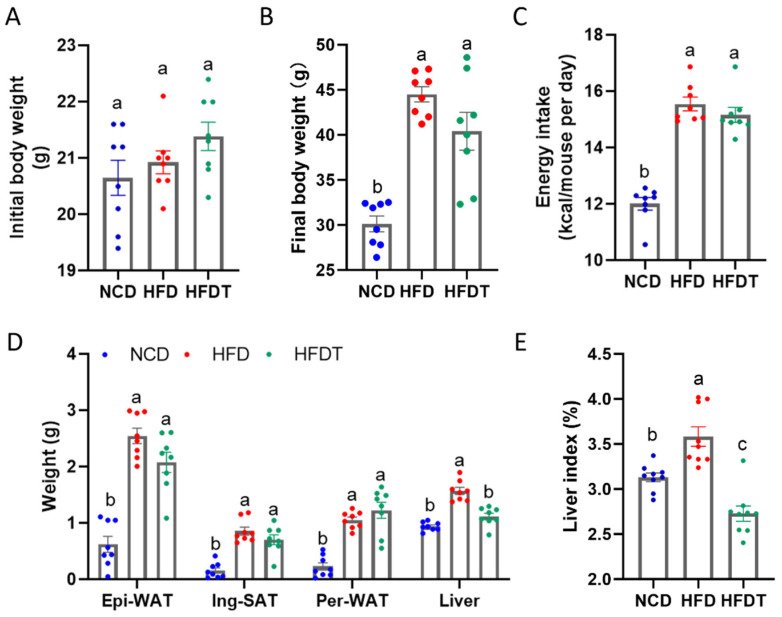
Effect of TP on body weight and liver index in HFD-fed mice. (**A**) Initial body weight; (**B**) Final body weight; (**C**) Energy intake; (**D**) Epi-WAT, Ing-SAT, Per-WAT and liver weight (**E**) Liver index. Values are expressed as the mean ± SEM. Graph bars in (**A**–**E**) marked with different letters represent statistically significant results (*p* < 0.05). (Abbreviated: Epi-WAT: Epididymal white adipose tissue; Ing-SAT: Inguinal subcutaneous adipose tissue; Per-WAT: Perirenal white adipose tissue).

We examined the phenotypes of TP treatment in HFD-fed mice for 15 weeks. As shown in Figure 2A, the initial body weight has no significant difference among the three groups. After 15 weeks of feeding, the final body weight, WAT weight and liver weight were significantly elevated in the HFD-fed mice compared to the NCD-fed mice (Figure 2B–E). TP supplementation did not affect the body weight but markedly decreased the liver weight and liver index in HFD-fed mice. The average energy intake of all the HFD-fed groups has no significant difference. 

### 3.2. TP Ameliorated Fat Accumulation and Hepatic Injury in HFD-Fed Mice

Hepatic steatosis is the physiological manifestation of obesity and non-alcoholic fatty liver disease. Thus, we measured the serum lipid levels to examine the antihyperlipidemic effect of TP. As shown in Table 1, compared to the NCD group, the contents of serum TG, TC and FFAs were markedly increased in HFD-fed mice and were decreased by TP administration. Other hepatic injury parameters such as high-density lipoprotein cholesterol (HDL-C), low-density lipoprotein cholesterol (LDL-C), alanine aminotransferase (ALT) and aspartate aminotransferase (AST) were also elevated in the HFD group compared to the NCD group. After TP treatment the content of ALT and AST were significantly decreased, further indicating the protective effect of TP on hepatic injury in HFD group. Moreover, the HFD feeding downregulated the content of serum AdipoQ, in contrast, the TP diet increased it in HFD-fed mice. Serum leptin levels had no obvious difference between HFD and HFDT. These results suggested that TP treatment ameliorated fat accumulation and hepatic injury in HFD-fed mice.

### 3.3. TP Improved Glucose Tolerance and Insulin Sensitivity in HFD-Fed Mice

**Figure 3:**
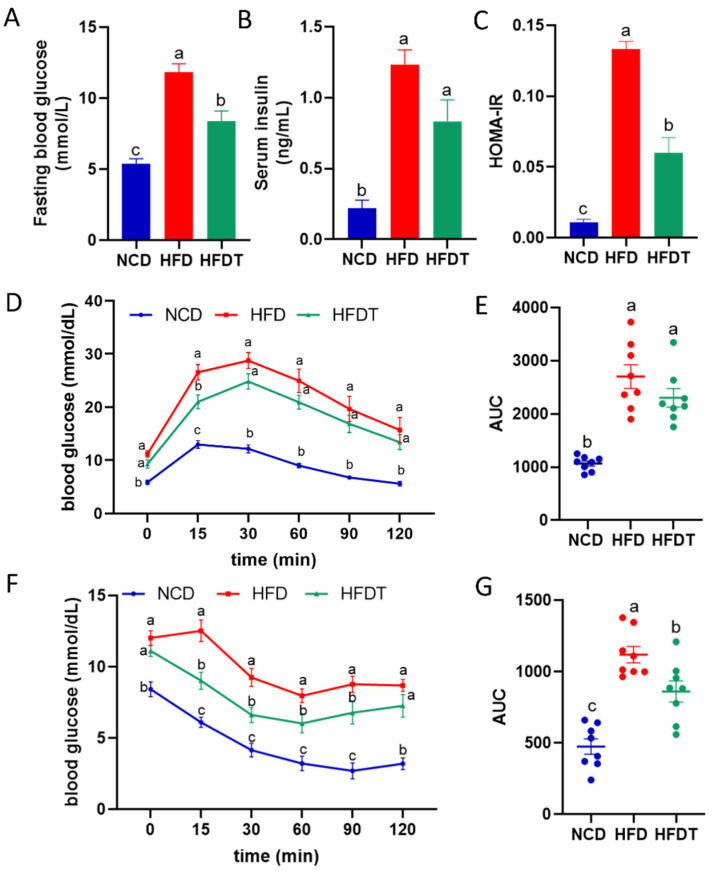
TP improved glucose tolerance and insulin sensitivity in HFD-fed mice. (**A**) Fasting blood glucose; (**B**) Serum insulin; (**C**) HOMA-IR; (**D**,**E**) ipGTT and AUC. (**F**,**G**) ITT and AUC. Values are expressed as the mean ± SEM. Graph bars in (**A**–**G**) marked with different letters represent statistically significant results (*p* < 0.05). (Abbreviated: HOMA-IR: Homeostasis model assessment-estimate insulin resistance; ipGTT: Intraperitoneal glucose tolerance test; ITT: Insulin tolerance test; AUC: Area under curve).

As chronic metabolic disease is tightly related with glucose tolerance and insulin resistance, we further tested fasting blood glucose and insulin levels. As shown in Figure 3A–C HFD mice showed increased blood glucose and insulin concentrations leading to increased levels of HOMA-IR, which demonstrated a higher degree of insulin resistance. TP reduced blood glucose and HOMA-IR in HFD group. Additionally, the serum glucose content from 0 min to 120 min in the ipGTT test was markedly higher in the HFD group than the NCD group (Figure 3D). TP treatment significantly decreased the glucose levels in 0 min and 15 min in HFD-fed mice. The AUC for the ipGTT measured in the HFDT and HFD group have no significant difference (Figure 3E). Similarly, the serum glucose content from 0 min to 120 min in the ITT test was markedly higher in the HFD group than in NCD group. TP treatment significantly decreased the glucose levels in 15, 30, 60 and 90 min in HFD-fed mice (Figure 3F). The AUC for ITT was also decreased in the HFDT group compared to the HFD group (Figure 3G). These results indicated that TP treatment prominently attenuated the impaired glucose tolerance and insulin resistance in HFD-fed mice.

### 3.4. TP Attenuated Hepatic Oxidative Stress and Inflammation in HFD-Fed Mice

**Figure 4:**
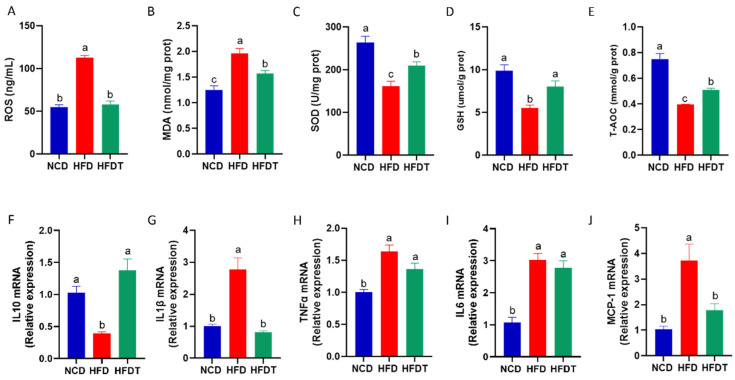
TP attenuated hepatic oxidative stress and inflammation in HFD-fed mice. (**A**–**E**) The concentration of ROS, MDA, SOD, GSH, T-AOC in liver; (**F**–**J**) The mRNA expression of IL10, IL1β, TNFα, IL6, MCP-1 in liver. Values are expressed as the mean ± SEM. Graph bars in (**A**–**J**) marked with different letters represent statistically significant results (*p* < 0.05). (Abbreviated: ROS: Reactive oxygen species; MDA: Malondialdehyde; SOD: Superoxide dismutase; GSH: Glutathione; T-AOC: Total antioxidant capacity; IL10: Interleukin-1β; IL1β: interleukin-1β; TNFα: Tumor necrosis factor-α; IL6: Interleukin-6; MCP-1: Monocyte chemoattractant protein-1).

Previous studies have shown that oxidative stress state plays a key role in the progress of insulin resistance. Our study further showed that 15 weeks of HFD feeding induced an increase in hepatic ROS levels, an indicator of oxidative stress (Figure 4A). TP treatment efficiently decreased the ROS levels to the normal content in the HFD group. Then we measured the hepatic MDA contents which was one oxidative damage product (Figure 4B). As shown in Figure 4A, TP administration significantly decreased the hepatic MDA content compared with HFD group. Additionally, we measured several key antioxidants, such as SOD, GSH and T-AOC (Figure 4C–E). We found that TP treatment significantly increased the activities of hepatic SOD, GSH and T-AOC in HFD-fed mice. Above all, these results showed an improved hepatic oxidative stress statement after TP administration in HFD-fed mice.

Increasing evidence indicated that chronic inflammation was strongly related to insulin resistance. In this study, we showed that the expression of proinflammatory cytokine interleukin 10 (IL10) was notably elevated in the HFD group compared to the NCD group, and TP treatment reversed the expression of IL10 in the liver (Figure 4F). Moreover, the expression of the anti-inflammatory cytokines, such as interleukin-1β (IL1β), TNFα, IL6 and Monocyte Chemoattractant Protein-1 (MCP-1) were markedly increased in HFD-fed mice (Figure 4G–J). TP administration efficiently decreased the expression of IL1β and MCP1. Thus, TP treatment attenuated the system inflammation in HFD-fed mice.

### 3.5. TP Enhanced Hepatic Insulin Signaling in HFD-Fed Mice

**Figure 5:**
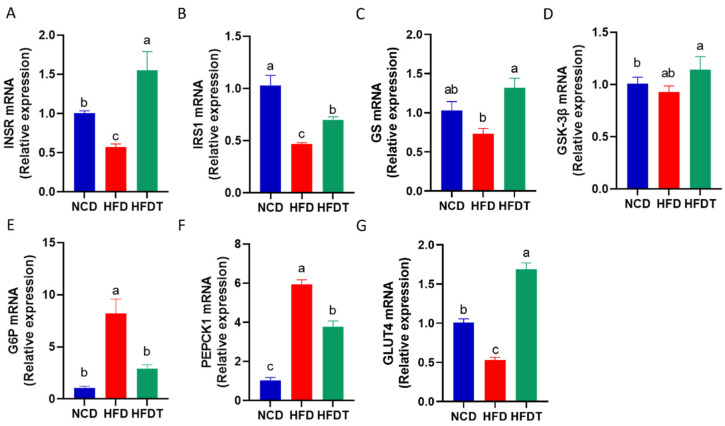
TP enhanced hepatic insulin signaling in HFD-fed mice. (**A**–**G**) The mRNA expression of INSR, IRS1, GS, GSK-3β, G6P, PEPCK1, GLUT4 in liver. Values are expressed as the mean ± SEM. Graph bars in (**A**–**G**) marked with different letters represent statistically significant results (*p* < 0.05). (Abbreviated: INSR: Insulin receptor; IRS1: Insulin receptor substrate-1; GS: Glycogen synthase; GSK-3β: Glycogen synthase kinase 3β; G6P: Glucose-6-phosphate; PEPCK1: Phosphoenolpyruvate carboxykinase 1; GLUT4: Glucose transporter type 4).

We further evaluated expression of genes involved in glucose metabolism. As shown in Figure 5, the insulin receptor and substrate genes, including insulin receptor (INSR) (Figure 5A) and IRS1 (Figure 5B) were decreased in HFD group compared to NCD group, whereas these trends were reversed by TP administration. Compared with HFD group, TP treatment had no significant effects on Glycogen synthase kinase 3β (GSK-3β), but increased the expression of glycogen synthase (GS) mRNA (Figure 5C,D). Additionally, the relative expression of glycogenolysis genes, including glucose-6-phosphatase (G6P) and phosphoenolpyruvate carboxykinase 1 (PEPCK1), was notably higher in the liver of the HFD group than in the NCD group (Figure 5E,F). TP supplementation attenuated the upregulation of G6P and PEPCK1. Moreover, the expression of GLUT4, a key regulator of glucose transport, was remarkably increased in the HFDT group compared to the HFD group (Figure 5G). 

**Figure 6:**
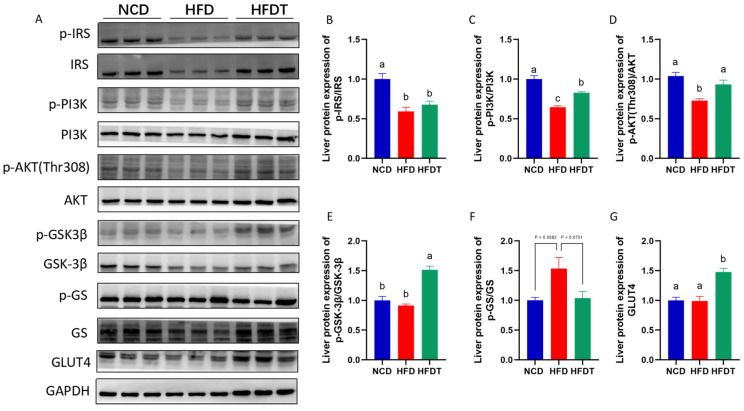
TP enhanced PI3K/AKT signaling in HFD-fed mice. (**A**–**G**) The protein levels of p-IRS/IRS, p-PI3K/PI3K, p-AKT/AKT, p-GSK-3β/GSK-3β, p-GS/GS, GLUT4 in liver. Values are expressed as the mean ± SEM. Graph bars in (**A**–**G**) marked with different letters represent statistically significant results (*p* < 0.05). (Abbreviated: p-IRS: Phospho-Insulin receptor substrate; IRS: Insulin receptor substrate; p-PI3K: Phospho-Phosphatidylinositol 3-kinase; PI3K: Phosphatidylinositol 3-kinase; p-AKT: Phospho-Protein Kinase B; AKT: Protein Kinase B; p-GSK-3β: Phospho-Glycogen synthase kinase 3β; GSK-3β: Glycogen synthase kinase 3β; p-GS: Phospho-Glycogen synthase; GS: Glycogen synthase; GLUT4: Glucose transporter type 4; GAPDH: Glyceraldehyde-3-phosphate dehydrogenase).

We next measured the protein expression of the insulin signaling pathway (Figure 6). Compared to NCD mice, HFD treatment significantly decreases the expression of p-IRS/IRS, p-PI3K/PI3K and p-AKT/AKT, and increases the expression of p-GS/GS with no significant difference (Figure 6A–D). TP treatment notably upregulated the level of p-PI3K/PI3K, p-AKT/AKT and p-GSK-3β/GSK-3β, and downregulated the level of p-GS/GS with no significant difference in HFD-fed mice (Figure 6A,E,F). Moreover, TP intervention significantly increased the expression of GLUT4 compared to the HFD group (Figure 6A,G). These results indicated that TP may repaired the destroyed hepatic insulin signaling pathway induced by HFD. 

## 4. Discussion

Controlling insulin resistance represents a viable strategy for enhancing glucose and lipid metabolism, thereby mitigating the risk of chronic metabolic disorders. Extensive research has investigated the interaction between natural products and chronic metabolic disease. Increasing evidence regarding the multifaceted biological functions of pectin, particularly apple pectin and citrus pectin have been deeply explored. TP is an active ingredient in tomato processing residue, but nevertheless the function of TP on chronic metabolic disease, especially on insulin resistance, inflammation and oxidative stress remains elusive. In this study, we elucidated that TP alleviated glucose metabolism by improving oxidative stress and inflammation through insulin signaling PI3K/AKT/GSK-3β pathway. Our findings provide experimental evidence to further explore innovative therapeutic strategies for preventing chronic metabolic disorders.

Based on the phenotypical analysis, we observed that TP treatment resulted in a significant reduction in body weight, liver weight, and liver index (Figure 2). Furthermore, TP administration effectively attenuated hepatic steatosis and injury in mice fed with an HFD. Consistently, TP supplementation markedly improved glucose tolerance and insulin tolerance which were impaired by HFD treatment (Figure 3). Hyperinsulinemia and hyperglycemia are characteristic features of insulin resistance and T2D [18]. Enhancing insulin resistance may represent a crucial strategy for the treatment or alleviation of chronic metabolic diseases. A previous study demonstrated that citrus pectin administration improved blood lipids levels, consistent with reduced insulin resistance [19]. Zammit et al. discovered that probiotics regulate lipid accumulation by enhancing insulin sensitivity, ultimately leading to decreased fat weight and other metabolic disorders [20]. In this study, fasting insulin, fasting blood glucose and HOMA-IR were elevated in the HFD group compared to the NCD group (Figure 3A,B). The therapeutic effect of TP treatment on glucose homeostasis is evidenced by lowering HOMA-IR, fasting insulin and glucose levels in agreement with improving ipGTT and ITT tests in HFD-fed mice. 

Oxidative stress and inflammation are typical pathophysiological responses associated with insulin resistance. The liver serves as the primary organ involved in detoxification processes; therefore, it is frequently exposed to damage caused by oxidative stress and inflammation. Clinical data and animal studies have shown an association between high levels of ROS and decreased insulin sensitivity in obese objects [11,21], which is consistent with our findings. Excessive ROS induces oxidative stress while promoting systemic inflammation, thereby accelerating an insulin resistant environment by inhibiting the phosphorylation of insulin receptors like IRS-1 and IRS-2 [22]. SOD and GSH being key enzymes responsible for scavenging ROS and protecting against hepatic injury induced by oxidative stress [16,23]. Additionally, MDA is a lipid peroxidation product and reflects the hepatic damage [16]. Our data found that TP notably elevated the activities of hepatic SOD, GSH and T-AOC levels and decreased the MDA content in HFD-fed mice. Moreover, the expression of anti-inflammatory cytokines, including IL1β, TNFα, IL6 and MCP1 were significantly decreased by TP treatment in HFD-fed mice. A previous study reported that increasing levels of TNFα and IL1β impairs the phosphorylation of IRS1/2 via severe mechanisms (MAPK, JNK and IKK) in obesity, resulting in insulin resistance in liver and adipose tissues [24]. These results note that TP treatment mitigates oxidative stress and inflammation, which could alleviate insulin resistance in HFD-fed mice. 

The PI3K/Akt signaling pathway plays a crucial role in hepatic insulin resistance by regulating glucose storage and synthesis through glycogenesis and glycogenolysis [25]. In this study, TP treatment enhanced AKT activation, which is a key component of the insulin signaling pathway. Dysregulated phosphorylation of AKT disrupted glucose and lipid metabolism, ultimately leading to insulin resistance and other pathological features [26]. GSK-3β serves as a primary substrate of the PI3K/AKT pathway in the regulation of glycogen synthesis, with its activity being inhibited by phosphorylation [27]. Our findings demonstrated that HFD treatment reduced mRNA levels of INSR, IRS1 and GS as well as protein levels of p-PI3K, p-AKT and p-GSK-3β but increased protein levels of p-GS. These effects were subsequently reversed by TP administration in HFD-fed mice. Furthermore, Akt activation upregulated the expression of glucose transporter GLUT4 while downregulating the expressions of the rate-limiting gluconeogenic enzymes of PEPCK and G6P, consistent with previous literature reports [28,29]. Collectively, these results indicate that TP can enhance insulin sensitivity and improve hepatic glucose metabolism by modulating the PI3K/AKT/GSK-3β pathway. 

## 5. Conclusions

TP treatment attenuated hepatic steatosis, enhanced glucose tolerance and insulin resistance and improved oxidative stress and inflammation. TP-regulated gene and protein expression in the PI3K/AKT/GSK-3β signaling pathway which might have contributed to improved glucose metabolism and ameliorated the insulin sensitivity in HFD-fed mice. Thus, our study provides a foundation for the further use of TP as a therapeutic agent against insulin resistance and related chronic metabolic disease. Further studies on human subjects are warranted to better assess the effects of long-term uses of TP against insulin resistance and related chronic metabolic disease. In addition, the evaluation of TP’s side effects and optimal dosage for humans will require clinical trials before it can become a routine therapy. These preliminary studies, however, do show promising efficacy of TP in the treatment against insulin resistance and related chronic metabolic disease. 

## Figures and Tables

**Table 1:** The serum biochemical parameters in NCD, HFD and HFDT groups.

Serum Parameters	NCD	HFD	HFDT
TG (mg/dL)	0.36 ± 0.02 ^c^	1.29 ± 0.09 ^a^	0.54 ± 0.07 ^b^
TC (mg/dL)	3.50 ± 0.19 ^c^	6.06 ± 0.29 ^a^	5.12 ± 0.19 ^b^
FFAs (μmol/L)	844.2 ± 11.68 ^c^	1655 ± 24.72 ^a^	1534 ± 39.75 ^b^
HDL-C (mmol/L)	1.77 ± 0.51 ^b^	4.19 ± 0.24 ^a^	3.71 ± 0.13 ^a^
LDL-C (mmol/L)	0.32 ± 0.08 ^b^	1.10 ± 0.23 ^a^	0.70 ± 0.09 ^ab^
ALT (U/L)	20.10 ± 4.51 ^b^	129.90 ± 53.12 ^a^	32.48 ± 4.73 ^b^
AST (U/L)	142.05 ± 16.26 ^b^	204.53 ± 26.29 ^a^	158.93 ± 33.35 ^b^
AdipoQ (ng/mL)	350.6 ± 6.65 ^a^	299.6 ± 1.82 ^b^	333.8 ± 5.46 ^a^
Leptin (ng/mL)	1.38 ± 0.02 ^b^	1.71 ± 0.04 ^a^	1.67 ± 0.02 ^a^

(Abbreviated: TG: Triacylglycerol; TC: Total cholesterol; FFAs: Free fatty acids; HDL-C: High-density lipoprotein cholesterol; LDL-C: Low-density lipoprotein cholesterol; ALT: Alanine aminotransferase; AST: Aspartate aminotransferase. Data are expressed as mean ± S.E.M. of 5 mice per group. a–c: means in the same column and in the same section having the same letters are not significantly different at *p* < 0.05).

## Data Availability

The data presented in this study are available on request from the corresponding author. The data are not publicly available due to privacy.

## Supplementary Materials

The following supporting information can be downloaded at: https://www.mdpi.com/article/10.3390/foods13030444/s1, Table S1: The composition of experimental diets; Table S2: Primary Antibodies for and WB.
