# Supplementary material for: Dietary Tomato Pectin Attenuates Hepatic Insulin Resistance and Inflammation in High-Fat-Diet Mice by Regulating the PI3K/AKT Pathway
Source: Foods. 2024 Jan 30;13(3):444. doi: 10.3390/foods13030444 (PMC10855921; doi:10.3390/foods13030444)
Supplement: Supplementary file 1 [file foods-13-00444-s001.zip › foods-2814217-supplementary.pdf]

Supplementary Table 1. The composition of experimental diets.

Ingredient	Normal control diet (NCD)		High-fat diet (HFD)	
	g%	kcal%	g%	kcal%
Casein, 30 Mesh	18.96	19.72	25.85	19.72
L-Cystine	0.28	0.30	0.39	0.30
Corn Starch	29.86	31.06	0.00	0.00
Maltodextrin 10	3.32	3.45	16.15	12.32
Sucrose	33.18	34.51	8.89	6.78
Cellulose, BW200	4.74	0	6.46	0.00
Soybean Oil	2.37	5.55	3.23	5.55
Lard	1.90	4.44	31.66	54.35
Mineral Mix S10026	0.95	0	1.29	0.00
DiCalcium Phosphate	1.23	0	1.68	0.00
Calcium Carbonate	0.52	0	0.71	0.00
Potassium Citrate, 1 H2O	1.56	0	2.13	0.00
Vitamin Mix V10001	0.95	1	1.29	0.99
Choline Bitartrate	0.19	0	0.26	0.00
Energy (kcal/g diet)	3.85		5.24	

Supplementary Table 2. Primary Antibodies for and WB

**WB**	**Concentration**	**Company**	**Catalogue No.**
GAPDH	1:1000	Cell Signaling Technology	5174
AKT	1:1000	Cell Signaling Technology	4691
Phospho-AKT(Ser473)	1:2000	Cell Signaling Technology	4060
GSK-3 β	1:1000	Cell Signaling Technology	9315
Phospho-GSK-3 β	1:1000	Cell Signaling Technology	9323
PI3K	1:1000	Cell Signaling Technology	4257
Phospho-PI3K	1:1000	Cell Signaling Technology	17366
GS	1:1000	Proteintech	10566-1-AP
Phospho-GS(Ser641)	1:1000	Cell Signaling Technology	47043
IRS	1:1000	Cell Signaling Technology	3407
Phospho-IRS	1:1000	Cell Signaling Technology	2385
GLUT4	1:1000	Proteintech	66846-1-lg
